# A Comprehensive Review on the Role of Melatonin's Anesthetic Applications in Pediatric Care

**DOI:** 10.7759/cureus.60575

**Published:** 2024-05-18

**Authors:** Sikha Subhadarshini, Karuna Taksande

**Affiliations:** 1 Anaesthesiology, Jawaharlal Nehru Medical College, Datta Meghe Institute of Higher Education and Research, Wardha, IND

**Keywords:** anesthesia, pediatric care, neuroprotection, analgesia, sedation, melatonin

## Abstract

Anesthesia is critical to pediatric care, ensuring the safety and comfort of children undergoing medical procedures. With a growing interest in alternative anesthetic agents, melatonin has emerged as a promising candidate due to its sedative, analgesic, anti-inflammatory, and neuroprotective properties. This comprehensive review explores the potential applications of melatonin in pediatric anesthesia. We delve into the pharmacological characteristics of melatonin, its anesthetic properties, and its clinical applications in pediatric care, including preoperative sedation, adjunct to general anesthesia, postoperative pain management, and prevention of emergence delirium. Additionally, we discuss the safety profile of melatonin, potential adverse effects, and comparative analysis with traditional anesthetics. Finally, we highlight future research directions to provide insights into melatonin's role in pediatric anesthesia and its implications for clinical practice.

## Introduction and background

Anesthesia plays a pivotal role in pediatric healthcare, ensuring the safe and comfortable execution of various medical procedures, including surgeries and diagnostic imaging. Due to children's unique physiological and psychological characteristics, tailored anesthetic approaches are necessary to achieve optimal outcomes while minimizing risks [1]. While traditional anesthetic agents have been effective, they have limitations and risks, particularly in pediatric populations. Hence, there has been a growing interest in exploring alternative agents that offer improved safety profiles, reduced side effects, and potentially enhanced efficacy [2]. Melatonin, primarily recognized for its role in regulating the sleep-wake cycle, has attracted attention for its pharmacological properties extending beyond chronobiotic effects. Emerging evidence indicates that melatonin possesses anesthetic properties, including sedative, analgesic, anti-inflammatory, and neuroprotective effects, making it a promising candidate for pediatric anesthesia [3]. This comprehensive review explores melatonin's potential applications in pediatric anesthesia. By synthesizing existing literature and clinical evidence, this review provides insights into melatonin's pharmacological characteristics, anesthetic properties, clinical applications in pediatric anesthesia, safety profile, comparative analysis with traditional anesthetics, and future research directions.

Melatonin's mechanisms of action involve binding to melatonin receptors, particularly melatonin receptor type 1A (MT1) and melatonin receptor type 2 (MT2), which are part of the G protein-coupled receptor (GPCR) family. This interaction influences physiological processes, including sleep regulation, circadian rhythms, and antioxidant functions [4]. Additionally, melatonin's anti-inflammatory and neuroprotective properties make it a multifaceted agent for pediatric anesthesia, potentially reducing perioperative complications and improving outcomes. While melatonin shows promise as an alternative anesthetic agent, further research is warranted to elucidate optimal dosing regimens, safety profiles in pediatric populations, and potential drug interactions. Comparative studies with traditional anesthetics are crucial for establishing melatonin's efficacy and safety. Moreover, exploring melatonin's role in specific pediatric populations and surgical procedures will provide valuable insights into its clinical applicability. This review aims to contribute to the growing body of knowledge on melatonin's potential in pediatric anesthesia and guide future research endeavors.

## Review

Methodology

The methodology employed for this review involved a comprehensive search of the literature to identify relevant studies on melatonin's potential applications in pediatric anesthesia. The search was conducted across multiple electronic databases, including PubMed, Scopus, Web of Science, and Google Scholar. The search strategy utilized a combination of keywords related to melatonin and pediatric anesthesia, such as "melatonin," "pediatric anesthesia," "anaesthetic properties," "sedative effects," "analgesic effects," "anti-inflammatory effects," and "neuroprotective effects." The search was not limited by publication date, and all types of studies, including clinical trials, observational studies, systematic reviews, and meta-analyses, were considered for inclusion. Additionally, the reference lists of relevant articles were manually searched to identify additional studies that met the inclusion criteria. The inclusion criteria encompassed studies that examined melatonin's pharmacological characteristics, anesthetic properties, clinical applications in pediatric anesthesia, safety profile, and comparative analysis with traditional anesthetics. Studies focusing on melatonin's use in pediatric populations and its potential efficacy and safety as an alternative anesthetic agent were prioritized. The search strategy aimed to comprehensively capture the available literature on melatonin's role in pediatric anesthesia to provide insights into its clinical applicability and guide future research directions.

Pharmacology of melatonin

Structure and Synthesis of Melatonin

Melatonin, a hormone synthesized in the pineal gland, retina, and brain, possesses a biochemical structure comprised of carbon, hydrogen, oxygen, and nitrogen molecules [4]. It boasts a molar mass of 232.283 g/mol and is classified within acetamide compounds [4]. The synthesis of melatonin follows the circadian rhythm, with peak production and release typically occurring at night [4]. Originating from the amino acid tryptophan, melatonin synthesis within the pineal gland involves a sequential enzymatic process converting tryptophan to serotonin and then to melatonin through the actions of serotonin N-acetyltransferase and acetylserotonin O-methyltransferase [5]. Melatonin's molecular weight is 232.28 g/mol, with a 1.175 g/cm^3^ density. Its melting point ranges between 116.5°C and 118°C, with a boiling point of 512.8°C [5]. Classified as an indolamine compound, melatonin features an indole chemical scaffold functionalized with 3-amide and 5-alkoxy groups [5]. These structural components, the 3-amide group and the 5-alkoxy group, confer amphiphilicity to the molecule, facilitating its passage across biological membranes and access to various cellular and subcellular compartments [5]. Beyond its biochemical properties, melatonin plays significant physiological roles in plants and animals. In plants, its biosynthesis initiates with the amino acid tryptophan, which plants can synthesize de novo via the shikimate pathway [6].

Mechanism of Action

Melatonin exerts its effects through binding to melatonin receptors, notably MT1 and MT2, both belonging to the GPCR family. As a potent full agonist, melatonin exhibits picomolar binding affinity for MT1 and nanomolar binding affinity for MT2. These receptors play pivotal roles in mediating melatonin's influence on various physiological processes, encompassing regulating sleep patterns, circadian rhythms, and antioxidant functions [7]. Upon binding to its receptors, melatonin modulates adenylate cyclase and inhibits cyclic adenosine monophosphate (cAMP) signaling pathways, thereby exerting its actions. Furthermore, within mitochondria, melatonin acts as a high-capacity antioxidant, counteracting free radicals and enhancing the expression of crucial antioxidant enzymes such as superoxide dismutase and glutathione peroxidase. This dual antioxidative role of melatonin is integral for cellular protection and overall health maintenance [8]. Additionally, melatonin's involvement in seasonal breeding mechanisms encompasses regulating seasonal fluctuations in sexual physiology by modulating the melatonin signal. In mammals with breeding seasons coinciding with longer daylight hours, melatonin can suppress libido by inhibiting the secretion of luteinizing hormone and follicle-stimulating hormone, thereby influencing reproductive functions [9].

Pharmacokinetics in the Pediatric Population

Pharmacokinetics in the pediatric population examines how drugs are absorbed, distributed, metabolized, and eliminated in children. This process can markedly differ from adults due to anatomical and physiological factors [10,11]. While children's doses are often determined based on age or weight, adjustments may be necessary considering individual differences in drug absorption, metabolism, and elimination rates [12]. The lack of pharmacokinetic studies in children presents challenges in establishing optimal dosing regimens. This often results in the extrapolation of doses from adult studies, which may only sometimes be appropriate for pediatric patients [10]. Addressing these challenges necessitates adopting model-based approaches, such as pharmacometrics, for designing dosing regimens and conducting clinical trials in pediatric pharmacology [13]. Successful pharmacokinetic research in children hinges upon establishing a robust infrastructure, securing sustainable funding, and fostering international collaborations to advance evidence-based pediatric pharmacotherapy [13].

Anesthetic properties of melatonin

Sedative Effects

Melatonin, a hormone synthesized by the pineal gland in the brain, plays a crucial role in regulating the body's circadian rhythm, which governs the sleep-wake cycle [14,15]. Supplements containing melatonin have been identified for their sedative effects, offering potential assistance to individuals experiencing disruptions in their circadian rhythms, such as those with jet lag or diminished melatonin levels, in achieving better sleep [14]. Scientific literature reviews indicate that melatonin supplements may aid in mitigating jet lag, particularly for individuals traversing five or more time zones. Moreover, they may outperform placebos by reducing sleep-onset time, extending total sleep duration, and enhancing daytime alertness in individuals grappling with insomnia [14]. Nevertheless, melatonin supplements carry certain risks. They may induce daytime drowsiness, stomach discomfort, dizziness, headaches, irritability, reduced libido, breast enlargement in males, and diminished sperm count [14]. Pregnant or nursing women are advised against taking melatonin supplements, and individuals with a history of hormonal-related issues should exercise caution and consult healthcare professionals before melatonin use [14]. Moreover, melatonin supplements have the potential to interact with various medications, including antidepressants, antipsychotics, benzodiazepines, and contraceptive drugs, and may exacerbate depressive symptoms in some individuals [8,14].

Analgesic Properties

Melatonin, a hormone secreted by the pineal gland, exhibits analgesic properties in both experimental and clinical settings. In animal studies, melatonin displays potent analgesic effects in a dose-dependent manner, effectively alleviating pain across various models, including electrically induced pain, thermally induced pain, hot-plate pain, neuropathic pain assessed through paw-withdrawal tests, and chemically induced pain [16,17]. These analgesic actions are believed to stem from melatonin's interaction with G(i)-coupled melatonin receptors, G(i)-coupled opioid μ-receptors, or gamma-aminobutyric acid type B (GABA-B) receptors, culminating in downstream alterations that reduce anxiety and mitigate pain sensations [16]. In clinical investigations, melatonin demonstrates analgesic benefits for individuals coping with chronic pain conditions such as fibromyalgia, irritable bowel syndrome, and migraine [16]. These therapeutic effects may be attributed to melatonin's ability to enhance sleep quality and alleviate anxiety, thereby contributing to diminished pain perception [16]. Furthermore, melatonin's anti-inflammatory properties have been explored, with evidence indicating its ability to mitigate inflammatory pain by inhibiting the production of nitric oxide (NO) via inducible NO synthase and modulating NO-cyclic guanosine monophosphate (cGMP) signaling pathways [17].

Anti-inflammatory Effects

Melatonin's anti-inflammatory properties hold promise for both the prevention and adjunctive treatment of inflammatory disorders. Systematic reviews of clinical trials have revealed that exogenous melatonin administration reduces inflammatory marker levels in humans, indicating its potential therapeutic utility in chronic inflammation management [18]. Across a spectrum of chronic diseases affecting various organs and contexts, including cardiovascular diseases, autoimmune disorders, metabolic disturbances, and psychiatric conditions, melatonin demonstrates anti-inflammatory activity [19]. By modulating the activation of the immune system and regulating the balance between pro- and anti-inflammatory molecules, melatonin effectively mitigates both chronic and acute inflammation [20]. Mechanistically, melatonin inhibits the expression of inflammatory mediators such as cyclooxygenase (COX) and inducible nitric oxide synthase (iNOS), pivotal players in the inflammatory cascade, while also curbing the production of other pro-inflammatory molecules like chemokines and adhesion molecules [20]. Furthermore, melatonin's inhibition of the Nod-like receptor protein 3 (NLRP3) inflammasome activation, which is involved in mounting inflammatory responses to pathogens and stressors, contributes to its anti-inflammatory effects [20]. With a favorable safety profile and minimal side effects, melatonin is a promising agent for preventing inflammatory disorders [20]. Its anti-inflammatory actions may be relevant in aging populations, where low-grade inflammation is prevalent [21]. Nonetheless, the outcomes of clinical trials investigating melatonin's anti-inflammatory effects have been inconsistent, underscoring the necessity for further research to establish its efficacy in this domain [19].

Neuroprotective Properties

Melatonin showcases notable neuroprotective effects across neurological disorders, spanning ischemia, Alzheimer's disease, and Parkinson's disease [22]. Its neuroprotective mechanisms primarily stem from its antioxidant properties, which shield brain cells against oxidative stress and damage induced by free radicals [22,23]. Evidence suggests that melatonin's ability to scavenge hydroxyl radicals may impede neurodegenerative processes involving free-radical generation and excitatory amino acid release [24]. Beyond its antioxidative attributes, melatonin exhibits anti-inflammatory, anti-excitotoxic, and anti-misfolding properties, further bolstering its neuroprotective role [22]. The capacity of melatonin to traverse the blood-brain barrier, coupled with its brief half-life and minimal side effects, positions it as a promising therapeutic candidate for neurological disorders [22]. Numerous studies have underscored the neuroprotective advantages of melatonin in experimental stroke models, showcasing reductions in infarct size and cerebral edema volume and the inhibition of caspase-3 activity in rodent brains [25]. Additionally, melatonin attenuates the cellular inflammatory response within the brain, safeguards against oxidative damage, and confers protection against grey and white matter injury, ultimately enhancing neuroplasticity and improving neurobehavioral and electrophysiological outcomes post-middle cerebral artery (MCA) occlusion in rodents [25].

Clinical applications in pediatric anesthesia

Preoperative Sedation

Preoperative sedation in pediatric patients stands as a standard practice geared towards alleviating preoperative anxiety and enhancing overall satisfaction with the anesthetic process. The benefits of sedative premedication include reducing both patient and parental apprehension, enhancing overall satisfaction, and facilitating the separation of the patient from their caregiver [26,27]. Decisions regarding preoperative tests hinge on factors such as the patient's clinical history, comorbidities, physical examination findings, and planned surgery [28]. The quest for the optimal route of premedication in children persists, with numerous studies exploring the pharmacokinetics, tolerability, efficacy, and safety of various formulations [28]. Among the commonly utilized sedative premedications in pediatric patients are midazolam, ketamine, and clonidine [27]. Midazolam, a benzodiazepine, imparts sedation, anxiolysis, and amnesia, whereas ketamine, an N-methyl-D-aspartate (NMDA) receptor antagonist, provides sedation, analgesia, and amnesia [27]. Clonidine, an alpha-2 adrenergic agonist, blunts the hemodynamic response during anesthetic induction and intubation, decreases the minimum alveolar concentration (MAC) for sevoflurane, and enhances postoperative analgesia [27].

Intranasal midazolam boasts a rapid onset of action, ease of administration, and high bioavailability. However, it may elicit stinging upon administration, and its duration of action might be shorter than oral midazolam [27]. Ketamine, administered intranasally, intramuscularly, or orally, offers a rapid onset of action but may trigger the emergence of delirium, nystagmus, and heightened postoperative nausea and vomiting [27]. Clonidine, available for oral, intranasal, or intramuscular administration, features a slower onset of action than midazolam, with a duration of action spanning two to four hours [27]. While the use of opiate medications in preoperative sedation is less common due to associated side effects like dysphoria, pre- and postoperative nausea, and vomiting, the preferred routes for administration typically include intranasal and oral, with fentanyl ranking as the most frequently employed drug [29].

Adjunct to General Anesthesia

As outlined in the enhanced recovery after surgery (ERAS) program, spinal anesthesia is a recommended adjunct to general anesthesia for laparoscopic colorectal surgery. This comprehensive approach involves the administration of various medications, including a carbohydrate-loaded drink, paracetamol, doxycycline, metronidazole, propofol, remifentanil, rocuronium, dexamethasone, ondansetron, bupivacaine, and fentanyl, among others, aimed at ensuring optimal patient care throughout the surgical procedure [30]. Remimazolam emerges as a viable adjunct to general anesthesia in pediatric patients, offering an alternative option for maintaining anesthesia during procedures. Extensive studies on a large cohort of cases have evaluated its adverse events and outcomes when utilized with general endotracheal anesthesia [31]. Lidocaine commonly serves as an adjunct to control intraoperative nociception and postoperative pain. Its infusion aids in pain management and reduces the requirement for opioids, contributing to a balanced approach to general anesthesia that seeks to maximize benefits while minimizing side effects [32]. Dexmedetomidine is administered as an adjunct to general anesthesia to provide anti-inflammatory effects. Research findings indicate that perioperative utilization of dexmedetomidine significantly decreases serum levels of inflammatory markers such as interleukin-6 (IL-6), interleukin-8 (IL-8), and tumor necrosis factor alpha (TNF-α), underscoring its potential to enhance patient outcomes during the perioperative period [33].

Postoperative Pain Management

Postoperative pain management in children is effectively addressed through a combination of analgesics, including acetaminophen (paracetamol), non-steroidal anti-inflammatory drugs (NSAIDs), opioids, and local/regional anesthesia [34]. Recent investigations have revealed that the dosage of acetaminophen necessary to achieve analgesia exceeds traditional dosages utilized for fever regulation: rectal administration of acetaminophen yields lower and more variable bioavailability than oral administration [34]. The utilization of NSAIDs in children is gaining momentum, with several studies demonstrating their robust analgesic potential [34]. Titration of opioids to the analgesic effect, alongside the adoption of nurse- and patient-controlled continuous opioid infusions, has become widespread and is regarded as an excellent approach to pain management in children [34]. Local peripheral and central blocks reduce the need for anesthetics during surgery and provide effective postoperative pain relief [34]. Regional anesthesia delivers superior postoperative analgesia and mitigates the stress response in infants and children [34]. The adoption of preventive strategies to manage pain whenever feasible, employing multi-modal analgesia, has proven efficacious across various scenarios, including day cases, significant surgeries, critically ill children, and infants [35]. Many acute pain services implement concurrent or co-analgesia techniques founded on four classes of analgesics: local anesthetics, opioids, NSAIDs, and acetaminophen (paracetamol) [35].

Prevention of Emergence Delirium (ED)

Prevention of ED in pediatric anesthesia is a paramount concern due to its potentially distressing symptoms and adverse outcomes for patients and caregivers. A systematic literature review has identified various interventions studied for this purpose [36]. One approach involves using midazolam, a benzodiazepine known for its anxiolytic and amnestic effects. A randomized study compared midazolam administered at induction versus 10 minutes before the end of surgery on ED incidence in children under sevoflurane anesthesia. Although both groups had similar ED incidences, the group receiving midazolam 10 minutes before surgery's end experienced significantly prolonged recovery [37]. Another study compared nalbuphine, a synthetic opioid agonist-antagonist, with ketamine's effects on ED incidence in children under sevoflurane anesthesia. Both nalbuphine and ketamine significantly reduced ED incidence compared to sevoflurane alone, with nalbuphine showing the highest benefit/risk ratio [38]. A multidisciplinary team at Pittsburgh VA developed a multicomponent intervention that has demonstrated efficacy in reducing ED occurrence and severity, along with related patient and staff injuries. This intervention involves routine risk factor screening, improved staff communication, environmental adjustments, specific medication strategies, and manual restraint application when necessary [36]. Pharmacologic regimens combining medications show more significant success in ED reduction than monotherapies. A meta-analysis has endorsed the addition of midazolam and antiemetics to enhance other medications' efficacy [39].

Potential Neuroprotective Effects During Surgery

The potential neuroprotective effects of various anesthetics during surgery have been extensively studied, revealing differing associations with neuroprotection. Intravenous anesthetics like thiopental, propofol, and etomidate have been linked to unproven neuroprotective efficacy [40]. However, lidocaine may exhibit neuroprotective properties in non-diabetic patients undergoing cardiac surgery with cardiopulmonary bypass, although conclusive evidence remains lacking [40]. The presumed mechanism of action for anesthetic neuroprotection involves enhancing neurological tissue tolerance to ischemia and modulating intracellular responses to energy supply deprivation [40]. Nonetheless, current evidence remains inconclusive, with some studies suggesting that intravenous anesthetics may possess both neuroprotective and neurotoxic attributes during the perioperative period [40]. The utilization of anesthetics with rapid elimination and minimal metabolic breakdown may contribute to reducing postoperative cognitive dysfunction in elderly surgical patients by facilitating quicker recovery from general anesthesia [41]. Likewise, employing shorter-acting anesthetic and analgesic agents could mitigate postoperative cognitive impairment in pediatric patients [41]. Neuroprotective strategies during cardiac surgery involve specific, receptor-mediated protective actions to safeguard the brain from neurological injury during and after surgery [42]. These encompass pharmacological and non-pharmacological interventions, all directed towards reducing neurological injury and enhancing patient outcomes [42]. Dexmedetomidine, a selective α2-adrenergic receptor agonist, has shown promise in mitigating surgery-related neuroinflammatory cascades and potentially exerting neuroprotective effects during cranial surgery [43]. In a double-blind, single-institution, randomized controlled trial, intraoperative dexmedetomidine infusion combined with goal-directed hemodynamic therapy (GDHT) during cranial surgery was associated with fewer patients experiencing postoperative neurological complications or postoperative delirium, without adverse hemodynamic effects [43].

Safety profile and adverse effects

Side Effects on the Pediatric Population

Melatonin is frequently used as a supplement to address sleep issues in children, yet its safety profile and potential adverse effects remain incompletely understood. Reported adverse effects in children often include nightmares, vivid dreams, and excessive sedation [44]. Despite its widespread use, the safety of melatonin in children has not been definitively established, necessitating further research to comprehend both short- and long-term effects in young individuals [45]. Limited research suggests potential short-term side effects such as headache, dizziness, drowsiness, agitation, and increased nighttime urination or bedwetting [45]. Concerns regarding melatonin's long-term effects on hormone levels and puberty have also been raised [45]. While melatonin use in children commonly results in non-serious adverse events, the true extent of such events and their long-term implications remain uncertain [46]. Given this knowledge gap, caution is advised against indiscriminate melatonin use in children and adolescents with insomnia [46]. Parents and caregivers must recognize that not all melatonin supplements are equivalent in purity and strength, with significant brand variability [47]. Although melatonin is generally considered safe for short-term use in adults, caution should be exercised for individuals with specific preexisting conditions or those taking certain medications [45]. Not everyone is suited for melatonin supplementation, and specific individuals should consult with a healthcare provider before initiating its use [45]. Careful consideration and consultation with a trusted healthcare provider are imperative before administering melatonin supplements to children [47].

Drug Interactions and Contraindications

Melatonin exhibits interactions with over 300 drugs, particularly those inducing drowsiness or dizziness, such as benzodiazepines, sedatives, hypnotics, sedating antihistamines, opioid analgesics, muscle relaxers, and certain herbs known to induce drowsiness [48]. Additionally, melatonin may heighten the risk of bleeding when taken alongside anticoagulants like warfarin, potentially affecting blood clotting [48]. Interaction with potent CYP1A2 inhibitors like zileuton may elevate melatonin levels or effects [49]. Moreover, melatonin may enhance the central nervous system effects of barbiturates and general anesthetics, potentially inducing relaxation and sleep [50]. Certain monoamine oxidase inhibitors (MAOIs), such as isocarboxazid (Marplan), phenelzine (Nardil), tranylcypromine (Parnate), and selegiline hydrochloride, as well as medications, like 5-hydroxytryptophan (5-HTP), amitriptyline, fluoxetine (Prozac), bupropion (Wellbutrin), and EFFEXOR® XR, can intensify anesthesia effects, particularly general anesthesia, necessitating discontinuation several weeks before surgery to mitigate potential cardiovascular effects [50]. Melatonin may also enhance the effectiveness of warfarin, heightening the risk of bruising and bleeding. Although interactions with birth control pills and caffeine have been noted, specific details are not provided [51]. Likewise, interactions with certain antidepressants and medications for lowering blood pressure have been suggested, emphasizing the need for caution and medical supervision when combining melatonin with these drugs [51]. Combining melatonin with sedative medications may lead to respiratory issues and excessive drowsiness due to compounded sedative effects [51].

Long-Term Safety Concerns

The long-term safety of melatonin usage still needs to be more adequately established, as there is a lack of published data on its safety beyond six months of daily administration [52]. Nevertheless, most studies suggest that melatonin is generally safe for short-term use in adults and children, with rare adverse effects reported [53,54]. Commonly reported adverse effects include mild symptoms such as daytime sleepiness, headaches, and dizziness, typically not significantly different from those observed with a placebo [53]. A recent systematic review encompassing various sleep and mental health disorders found only four instances of individuals discontinuing participation in the included trials due to side effects, with headaches being the most frequently reported [53]. Further, a narrative review echoed similar findings, highlighting daytime sleepiness, headaches, and dizziness as the most common adverse effects. However, most studies concluded that these effects did not significantly differ from those observed with a placebo, and the reported symptoms were of minor clinical significance [53]. Despite these findings, potential long-term adverse effects of melatonin use have been identified, including the inhibition of reproductive function, delayed puberty timing, and potential impacts on fetal and neonatal circadian rhythms when used during pregnancy and lactation [52]. Moreover, the interactions between melatonin and other medications remain largely unexplored, and there is insufficient data regarding its use in organic or psychiatric diseases for comprehensive evaluation [52]. Consequently, while melatonin is generally considered safe for short-term usage, its long-term safety profile remains uncertain, necessitating further research to ascertain its safety and efficacy for prolonged use [52,53].

Comparative analysis with traditional anesthetics

Efficacy Compared to Benzodiazepines

Melatonin has emerged as a promising alternative to alprazolam for premedication in alleviating anxiety among patients undergoing surgery [55]. A comparative study investigating the effects of oral melatonin (6 mg) versus oral alprazolam (0.5 mg) on preoperative anxiety, sedation, and cognitive and psychomotor functions demonstrated melatonin's superiority as an anxiolytic over alprazolam. Melatonin provided significant anxiolysis and sedation 60-120 minutes before surgery, maintaining stable hemodynamics and inducing minimal side effects [55]. Importantly, melatonin was observed to lack anterograde amnesia, a desirable characteristic for premedication use [55]. With its broad therapeutic index and low incidence of side effects, melatonin presents a favorable alternative to benzodiazepines and similar hypnotic drugs [56]. Clinical trials have further explored melatonin's efficacy in addressing sleep disturbances among individuals receiving methadone maintenance and utilizing benzodiazepines. In one trial, melatonin improved sleep efficiency among 19 patients with schizophrenia experiencing poor sleep quality [57]. Another randomized clinical trial revealed that melatonin not only enhanced sleep quality in patients unable to discontinue benzodiazepines but also prolonged the time to relapse in those who successfully ceased benzodiazepine use [58]. If replicated, these findings suggest the potential utility of melatonin in managing sleep difficulties in patients undergoing methadone maintenance and utilizing anxiolytics, as well as serving as an adjunct to reduce benzodiazepine relapse [57,58].

Safety Profile Compared to Opioids

In clinical practice, comparing the safety profiles of melatonin to opioids such as morphine is crucial. Melatonin exhibits a notably favorable safety profile, rendering it a potentially preferable choice for specific applications, notably insomnia in medical inpatients [59]. Conversely, opioids like morphine, while potent analgesics, are accompanied by well-documented side effects, including dependence, addiction, respiratory depression, and constipation, which can restrict their clinical utility [60]. In terms of safety, melatonin is generally well-tolerated and associated with fewer side effects compared to opioids such as morphine. Melatonin supplementation carries a lower risk of adverse effects, positioning it as a safer alternative for specific patient populations, particularly in addressing sleep disorders and regulating circadian rhythms [59]. Conversely, opioids, including morphine, are notorious for their higher potential for dependence, addiction, and respiratory depression, posing significant risks, especially in cases of prolonged use or high doses [60].

Cost-Effectiveness and Availability

Melatonin is a widely accessible over-the-counter dietary supplement regulated by the Food and Drug Administration (FDA), commonly utilized therapeutically for insomnia in adults and primary sleep disorders in children [61,62]. Available in various formulations like tablets, capsules, liquids, and gummies, melatonin offers a cost-effective alternative to traditional anesthetics [61,62]. In pediatric patients, melatonin demonstrates efficacy in alleviating preoperative anxiety and inducing significant sedation, rendering it a potential candidate for sedation purposes and facilitating various medical procedures, including venipuncture and mask application during anesthesia induction [63]. However, establishing a consensus on the ideal melatonin dosage for pediatric sedation still needs to be discovered, with reported dosages spanning from 0.3 to 20 mg [63]. While generally well-tolerated with short-term use, melatonin may elicit diverse albeit infrequent adverse effects, predominantly mild to moderate severity [51]. Patients with hepatic impairment should avoid melatonin due to reduced clearance, and caution is warranted regarding potential interactions with medications metabolized by CYP1A enzymes [51]. Despite its cost-effectiveness and availability, further research is imperative to delineate the toxicity and outcomes associated with melatonin ingestion in children, as over-the-counter melatonin usage may engender potential adverse events [62]. Public health initiatives should prioritize raising awareness of escalating melatonin ingestion among children and ensuring the quality and safety of melatonin products.

## Conclusions

Melatonin emerges as a promising alternative anesthetic agent in pediatric care, offering a range of sedative, analgesic, anti-inflammatory, and neuroprotective properties. Its potential applications span from preoperative sedation to postoperative pain management, presenting a versatile option for various stages of anesthesia. With its favorable safety profile and potential to mitigate the adverse effects associated with traditional anesthetics, melatonin holds significant implications for clinical practice. Integrating melatonin into pediatric anesthesia protocols could enhance patient comfort, reduce reliance on conventional anesthetics, and improve postoperative outcomes.
